# Comprehensive analysis of predictors and outcomes following Vibrant Soundbridge implantation – part 1 of a prospective study

**DOI:** 10.1038/s41598-025-20966-y

**Published:** 2025-10-10

**Authors:** Christoph Müller, Hannes Seidler, Anna Tsypina, Janina Kuch, Thomas Zahnert, Susen Lailach

**Affiliations:** 1 Ear Research Center Dresden, Department of Otorhinolaryngology, Head and Neck Surgery, TU Dresden, Medical Faculty and University Hospital Carl Gustav Carus, Dresden, Germany; 2 Carl Gustav Carus Faculty of Medicine, TU Dresden, Dresden, Germany

**Keywords:** Active middle ear implant, AMEI, Vibrant Soundbridge, LLM prediction, Outcome parameter, Speech intelligibility, Outcomes research, Sensory systems, Transduction

## Abstract

Active middle ear implants such as the Vibrant Soundbridge (VSB) represent effective treatment options for patients with conductive or mixed hearing loss when conventional hearing aids are insufficient. Nevertheless, postoperative speech intelligibility varies considerably, even among patients with comparable audiometric profiles. This first part of a two-part prospective observational study aimed to analyze outcomes and identify clinically accessible predictors of postoperative speech intelligibility. Twenty adults who underwent unilateral VSB implantation were included. Audiological assessments comprised postoperative bone conduction (BC), Vibrogram (VIB), free-field thresholds, maximum unaided speech intelligibility (WRS_max_), and aided speech intelligibility at 65 dB SPL (WRS_65dB_). Additional parameters included coupling efficiency, effective gain, dynamic range, loudness scaling, and patient-reported outcomes (PROMs; IOI-HA, SSQ12). Key predictors of WRS_65dB_ were identified using Spearman correlation analysis (ρ). The mean postoperative WRS_65dB_ was 81.5% (SD = 9.0%). The strongest correlations were observed with age (ρ = − 0.63) and two surrogate parameters of cochlear reserve - WRS_max_ (ρ = 0.56) and postoperative BC_PTA4_ (ρ = − 0.52). PROMs confirmed high subjective benefit and displayed correlations with audiological outcomes. Age, WRS_max_ and postoperative BC_PTA4_ will serve as the basis for the AI-based prediction model presented in part II. The correlation of PROMs with audiological outcomes highlights the importance of integrating objective and subjective measures in hearing rehabilitation.

**Supplementary Information:**

The online version contains supplementary material available at 10.1038/s41598-025-20966-y.

## Introduction

Active middle ear implants (AMEI) have become an integral part of concepts for the rehabilitation of mixed (MHL) or conductive (CHL) hearing loss, especially when conventional hearing aids (CHA) are limited in patients with a high air-bone gap due to the required amplification power and output level. The Vibrant Soundbridge (VSB, MED-EL, Innsbruck, Austria) is currently the only AMEI available internationally. However, the results of VSB implantation are not achieved in the same way for all patients. Understanding the factors that influence the outcomes of VSB implantation is crucial for optimizing patient selection, surgical techniques, and postoperative treatment process.

The preoperative bone conduction (BC) hearing threshold has already been discussed as an important factor. Extensive experimental studies and calculations indicate that the indication limits propagated by the manufacturer are often very optimistic. Taking into account a dynamic range of 35 dB, the maximum averaged BC hearing threshold to be recommended for VSB implantation is 47 dB, according to the studies by Rahne and Plontke^1^. However, even in this very precise indication range, the speech intelligibility results obtained postoperatively are highly scattered. Sometimes, in individual examinations, only a speech intelligibility of lower than 60% at 65 dB SPL is achieved for individual patients, which may already be an indication criteria for cochlear implantation^2–4^. However, even with audiometrically identical preoperative BC thresholds, speech intelligibility with the VSB can vary widely, which can make patient counselling and selection very complex, especially in the overlapping indication range for cochlear implantation^2,5,6^.

However, the quality of the coupling of the actor (floating mass transducer, FMT) also has a significant influence on the postoperative results. Müller et al. were able to demonstrate that a satisfactory speech intelligibility of 75% can be achieved with a coupling loss of less than 20 dB^6^. The coupling quality (‘coupling efficiency’ (CE)) is measured by the difference between the in-situ thresholds (Vibrogram threshold) and BC thresholds. The highest coupling deficits for the FMT occur in the low-frequency range around 0.5 kHz and are most pronounced when comparing different coupling modalities for the round window membrane^7^. The CE is essentially based on the anatomical conditions, the optimization of the coupling elements by the manufacturer and, above all, the expertise of the surgeon. The current attempts to improve coupling using objective intraoperative measurement procedures are therefore reasonable^8^. However, as can be seen in the publication by Müller et al. and observed in everyday clinical practice, speech intelligibility can vary considerably even with the same coupling quality^6^.

Furthermore, the fitting quality of the systems is of particular importance. To date, however, there is no consistent standard for describing the fitting quality in AMEI outcome reports^9^. However, near-threshold measurements (free-field (FF) threshold) for CHAs are considered inappropriate for assessing the fitting quality. The measurement of the aided threshold in the free-field makes it possible to estimate the lowest input level that is audible to the hearing aid user. This information makes the aided threshold a useful index reflecting the goodness of fit of a non-linear CHA for low input levels^10^. However, the aided threshold does not provide any information about the transmission behavior of medium and loud signals and does not allow any conclusions to be drawn about the speech intelligibility achieved with the AMEI, but only documents the shift of the hearing threshold into the speech-audible range^11^. Accordingly, a discrepancy between the frequency-weighted FF threshold (articulation index) and speech intelligibility was also shown in clinical studies on the quality of results of fitting with AMEI^12^. The concept of the articulation index was initially developed for the fitting control of linear hearing systems. Due to multiple interfering factors (including speech and noise spectra, reverberation, masking effects, distortions) and the associated inadequate prediction of speech intelligibility, this index currently only plays a subordinate role^13^.

In contrast to the FF threshold, the indication of above-threshold tests such as loudness scaling can contribute to adequate documentation of the fitting quality of implantable hearing systems^9^. It is reasonable to assume that some of the variability in treatment results may also be due to a potentially optimizable fitting of the system, particularly in the range of near-threshold loudness.

This manuscript (Part 1 of a two-part publication series) aims to provide a comprehensive analysis of these influencing factors, drawing on recent research and clinical findings. By identifying and understanding the predictors of successful outcomes, we can enhance the efficacy of VSB implantation and improve the quality of life for individuals with hearing impairments. In the second part, an AI-based model will be developed and presented to predict postoperative outcomes—particularly speech intelligibility—based on the data introduced in this manuscript.

## Results

Data of 20 patients (sex: 10 female, 10 male patients; age: 47.0 ± 16.1 (SD) years; unaided WRS_max_: 88.0 ± 12.3 (SD) %, minimum: 60%, maximum: 100%; aided WRS_65dB_: 81.5 ± 9.0 (SD) %, minimum: 60% (low performer ID 20), maximum: 95%) could be evaluated. Please refer to Table 1 for information such as demographic details, etiology of hearing loss, addressed coupling site and applied coupler. In 8 out of 20 patients the cause of hearing loss was chronic otitis media without cholesteatoma (COMsC), in 11 patients it was chronic otitis media with cholesteatoma (COMcC). In 13 of 20 patients the round window was chosen as the coupling site, 10 of these 13 patients were implanted with a round window soft coupler (RWSC), 3 received a round window titanium coupler (RWTC).

**Table 1:** Details regarding the implanted patients. * The time of the calculation is referenced to the date of the study measurements. COMsC (chronic otitis media without cholesteatoma), COMcC (chronic otitis media with cholesteatoma), round window (RW), oval window (OW), SP (short process), RWTC (round window titanium coupler), RWSC (round window soft coupler), sclip (stapes clip coupler), SSH (stapes SH coupler), OWC (oval window coupler), SP (incus short process coupler).

Item Patient ID	Age	Time since VSB implantation in months*	Etiology of hearing loss	Coupling site	Coupler
1 (mild outlier in BC_PTA4_)	78	6	COMsC, multiple ear surgeries	RW	RWTC
2	58	12	COMsC, tympanosclerosis	RW	RWSC
3	30	11	COMcC, multiple ear surgeries, radical cavity, external ear canal stenosis	RW	RWSC
4 (mild outlier in CE_PTA4_)	36	7	external ear canal atresia, stapes fixation	RW	RWSC
5	67	11	COMsC, multiple ear surgeries, tympanosclerosis	RW	RWTC
6	57	40	COMcC, multiple ear surgeries, radical cavity	RW	RWSC
7	35	23	COMsC, condition after TBC with labyrinthitis	OW	OWC
8	41	46	COMcC, multiple ear surgeries, radical cavity	RW	RWSC
9	33	15	COMsC, multiple ear surgeries	Stapes	SClip
10	61	49	COMcC, multiple ear surgeries, radical cavity	Stapes	SClip
11	32	43	COMsC, condition after stapes surgery in external hospital	RW	RWSC
12	27	25	COMcC, multiple ear surgeries, radical cavity, condition after flooting footplate	RW	RWSC
13	30	105	COMcC, multiple ear surgeries, change from BB to VSB due to recurrent headaches	RW	RWSC
14	46	37	COMcC, multiple ear surgeries, wet radical cavity	OW	OWC
15	69	48	COMcC, multiple ear surgeries, radical cavity, post-inflammatory stapes fixation	RW	RWSC
16	54	107	COMcC, multiple ear surgeries, radical cavity	RW	RWSC
17	37	3	COMcC, multiple ear surgeries, radical cavity, external ear canal stenosis	Stapes	SSH
18 (strong outlier in EG_PTA4_)	28	3	COMcC, multiple ear surgeries, radical cavity	OW	OWC
19	53	3	COMsC, condition after release of malleus head fixation	Incus SP	SPC
20 (low performer)	68	5	COMsC, tympanosclerosis, post-inflammatory stapes fixation	RW	RWTC

### Threshold based measurements

**Fig. 1:**
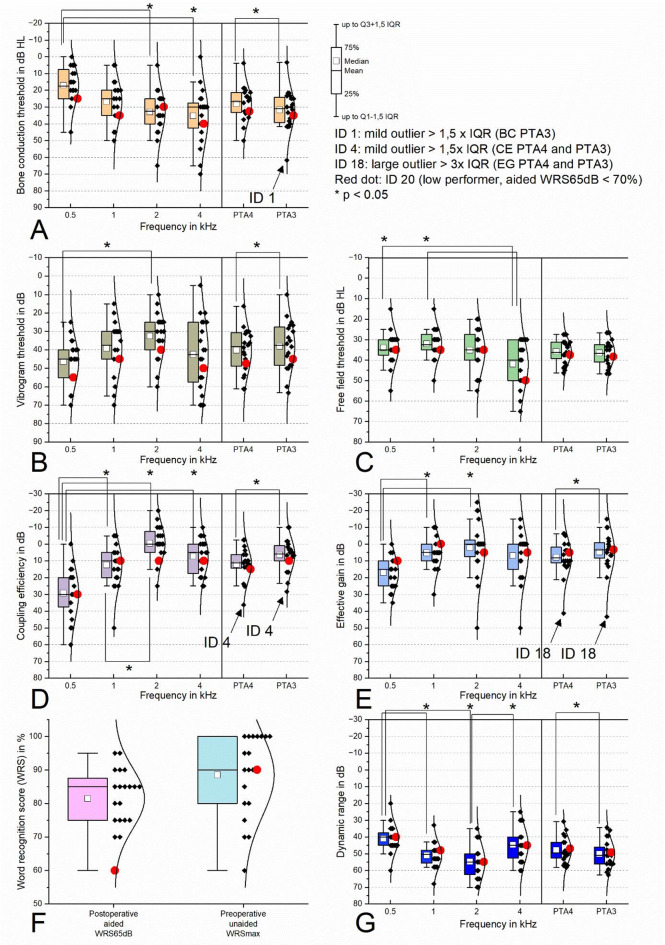
(**A**) **Bone conduction (BC) thresholds**: significant differences were found between 0.5 and 2 kHz, 0.5 and 4 kHz, and between PTA4 and PTA3. Please note the mild outlier (ID 1). (**B**) **Vibrogram (VIB) thresholds**: significant differences were observed between 0.5 and 2 kHz and between PTA4 and PTA3. (**C**) **Free-field measurements**: significant differences were found between 0.5 and 4 kHz, and between 1 and 4 kHz; no significant differences were observed between PTA4 and PTA3. (**D**) **Coupling efficiency (CE)**: significant differences occurred between 0.5 and 1 kHz, 0.5 and 2, and 0.5 and 4 kHz, as well as between PTA4 and PTA3. Please note the mild outlier (ID 4). (**E**) **Effective gain (EG)**: significant differences were found between 0.5 and 1 kHz, 0.5 and 2 kHz, and between PTA4 and PTA3. Please note the large outlier (ID 18). (**F**) **Postoperative aided WRS**_65dB_ **and** **preoperative unaided WRS**_max_. (**G**) **Dynamic range (DR)**: significant differences were observed between 0.5 and 1 kHz, 0.5 and 2 kHz, 2 and 4 kHz, and between PTA4 and PTA3. Please note the low performer (ID 20, red dot) visible in all subgraphics. * Significant results, *p* < 0.05.

The results of the threshold-based measurements (Fig. 1) are summarized in Table 2. When comparing the means of PTA4 and PTA3, significant differences (*p* < 0.05) were observed for BC, Vibrogram, coupling efficiency, and dynamic range, with effect sizes ranging from medium (VIB) to large (BC_PTA4_, CE, EG, DR) in the paired t-tests. No significant differences were found for free-field measurements, which showed only a small effect size. At the level of individual frequencies, significant differences were detected across all parameters and between at least one pair of frequencies, with effect sizes ranging from moderate (VIB, FF) to large (BC, CE, EG, DR) based on ANOVA. Across all parameters, 0.5 kHz was most frequently affected and differed significantly from at least one other frequency. As the results for unaided WRS_max_ and aided WRS_65dB_ were reported in the preceding paragraph, they are not reiterated here. Mild outliers were found in BC_PTA4_ (ID 1) and CE_PTA4_ (ID 4), strong outliers in EG_PTA4_ (ID 18).

**Table 2:** Summary of results from threshold-based measurements: paired t-tests revealed significant differences between PTA4 and PTA3 for BC, VIB, CE, EG, and DR, with effect sizes ranging from medium (VIB) to large (BC, FF, CE, EG, DR). ANOVA showed moderate (VIB, FF) to large (BC, CE, EG, DR) effect sizes, with most significant differences found between the mean at 0.5 kHz and other frequencies. Bone conduction (BC), vibrogram (VIB), free-field (FF), coupling efficiency (CE), effective gain (EG), dynamic range (DR).

	BC (Fig. 1A)		VIB (Fig. 1B)		FF (Fig. 1C)		CE (Fig. 1D)		EG (Fig. 1E)		DR (Fig. 1G)
Differences between PTA4 and PTA3 (paired t-test)											
Mean PTA4 ± SD in dB	28.0 ± 10.1		40.2 ± 11.9		35.8 ± 5.8		12.1 ± 9.6		7.8 ± 10.6		47.8 ± 7.4
Mean PTA3 ± SD in dB	31.7 ± 11.7		38.1 ± 13.3		36.4 ± 6.4		6.4 ± 12.1		4.8 ± 12.3		50.2 ± 8.1
p-value (paired t-test)	< 0.001		0.003		0.104		< 0.001		< 0.001		< 0.001
Interpretation of p-value	significant		significant		not significant		significant		significant		significant
|g*| (effect size t-test)	1.1		0.7		0.3		1.7		1.0		1.2
Interpretation of |g*|	large		medium		small		large		large		large
Differences between the individual frequencies (univariate ANOVA)											
Mean 0.5 kHz in dB	17.0 ± 10.8	46.5 ± 11.9		34.0 ± 8.0		29.3 ± 14.7		17.0 ± 9.7		41.0 ± 8.0	
Mean 1 kHz in dB	33.0 ± 12.8	39.3 ± 14.7		32.0 ± 7.3		12.5 ± 13.1		5.25 ± 9.8		51.0 ± 7.3	
Mean 2 kHz in dB	26.8 ± 12.4	32.5 ± 12.5		35.3 ± 10.1		0.5 ± 10.5		2.25 ± 16.8		54.8 ± 10.1	
Mean 4 kHz in dB	35.3 ± 16.4	42.5 ± 19.2		42.0 ± 11.2		7.25 ± 10.7		6.75 ± 14.53		44.8 ± 10.1	
ω² (effect size of ANOVA)	0.20	0.08		0.11		0.43		0.13		0.24	
Interpretation of ω²	large	moderate		moderate		large		large		large	
Post-hoc Tukey test < 0.05 between the individual frequencies	0.5 and 2 kHz 0.5 and 4 kHz	0.5 and 2 kHz		0.5 and 4 kHz 1 and 4 kHz		0.5 and 1 kHz 0.5 and 2 kHz 0.5 and 4 kHz		0.5 and 1 kHz 0.5 and 2 kHz		0.5 and 1 kHz 0.5 and 2 kHz 2 and 4 kHz	

### Correlation of threshold based measurements and speech intelligibility

**Fig. 2:**
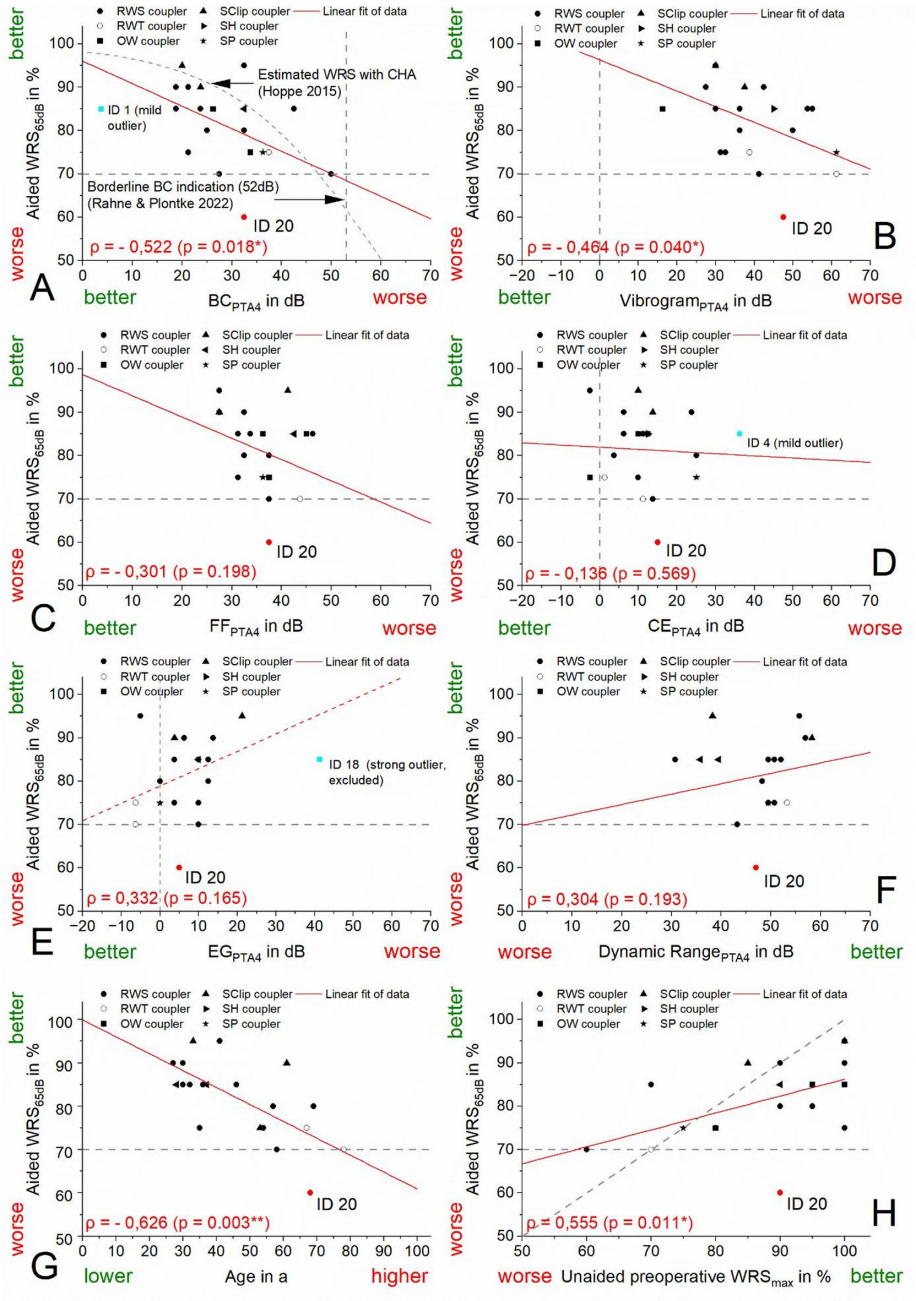
Correlations of threshold based predictive parameters (**A**: BC_PTA4_, **B**: VIB_PTA4_, **C**: FF_PTA4_, **D**: CE_PTA4,_ **E**: EG_PTA4_, **F**: DR_PTA4_) and additional non-threshold-based parameters (**G**: age, **H**: unaided preoperative WRS_max_) with postoperative aided speech intelligibility (WRS_65dB_; dashed line at 70% indicates cut-of between high and low performance). Strong significant correlations (*| ρ |* > 0.5, *p* < 0.05) for BC_PTA4_, age, unaided preoperative WRS_max_. Moderate significant correlations (0.3 < *| ρ |* < 0.5, *p* < 0.05) for VIB. Moderate but non-significant correlations (0.3 < *| ρ |* < 0.5, *p* > 0.05) for FF, EG, DR. Weak non-significant correlation (0.1 < *| ρ |* < 0.3, *p* < 0.05) for CE_PTA4_. Data of Hoppe et al. 2015 and Rahne and Plontke et al. 2022 are added to Fig A.

The correlations between the PTA4 thresholds of BC, VIB, FF, CE, EG, and DR (Supp. File 2, Table A6) and the postoperative aided WRS_65dB_ were strong (BC_PTA4_, ρ = −0.522, *p* < 0.05), moderate (VIB, ρ = −0.464, *p* < 0.05; FF, ρ = −0.301, *p* > 0.05; EG, ρ = 0.336, *p* > 0.05; DR, ρ = 0.304, *p* > 0.05), or weak (CE, ρ = −0.136, *p* > 0.05), and were graphically illustrated (Fig. 2) using linear fit functions (Supp. 2, Table A7). Correlations were also calculated for the PTA3 thresholds of these parameters (Supp. 2, Table A7); however, they were consistently lower and showed reduced or absent statistical significance. Therefore, only the PTA4 thresholds were selected for further use in the prediction model.

Three additional parameters were analyzed for their correlation with the aided WRS_65dB_: WRS_max_, age and difference of preoperative unaided WRS_max_ and the postoperative aided WRS_65dB_. WRS_max_ and age showed a strong correlation (ρ = 0.555, *p* < 0.05 (WRS_max_), ρ = −0.626, *p* < 0.05 (age)) with postoperative aided WRS_65dB_. The difference between the preoperative unaided WRS_max_ and the postoperative aided WRS_65dB_ (ρ = −0.111, *p*> 0.05, Supp. File 2, Table A6), which had been highlighted by Müller et al.^6^ as a key predictor of postoperative speech understanding, did not demonstrate satisfactory results in our cohort.

### Categorical loudness scaling

**Fig. 3:**
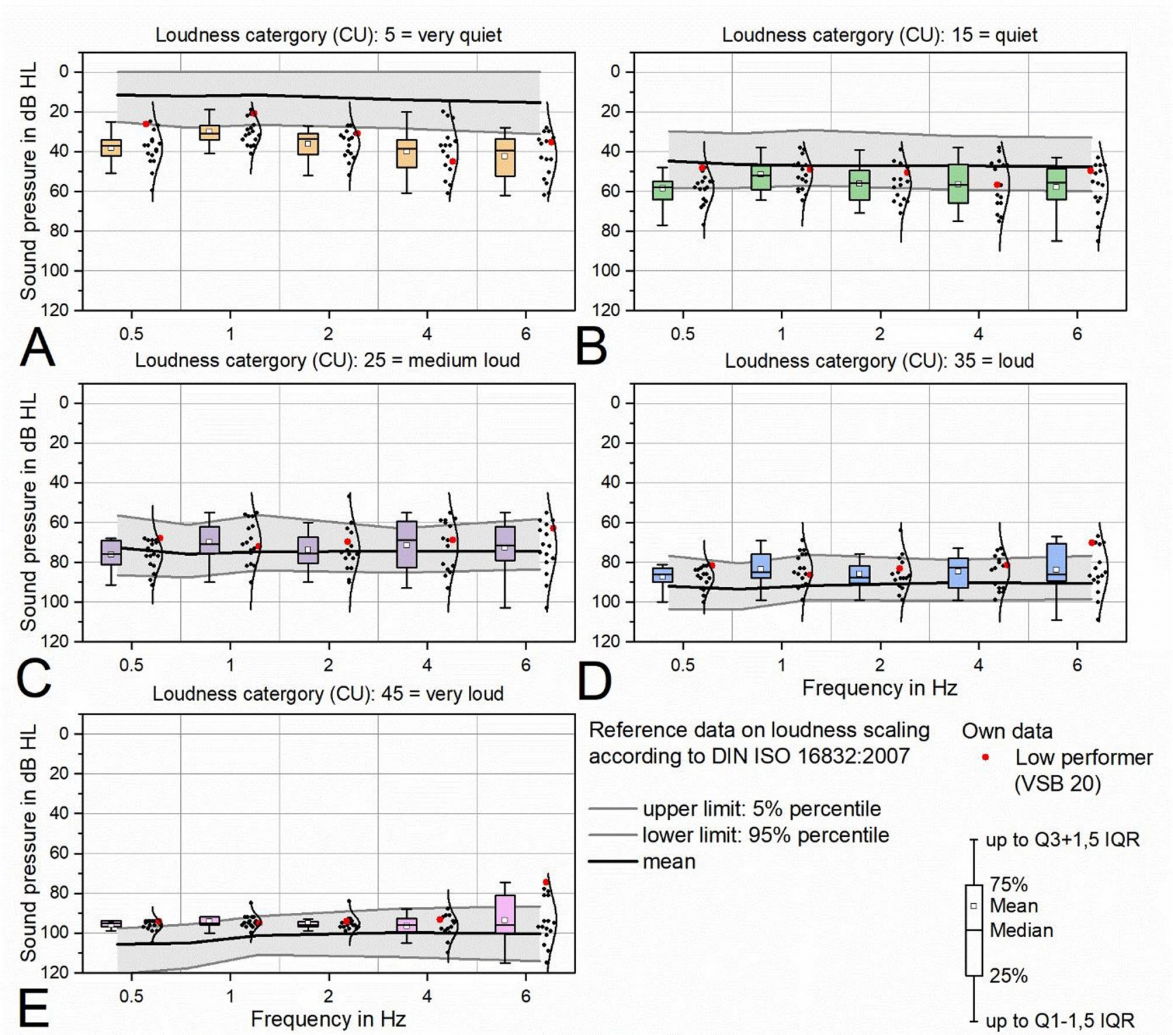
Categorical loudness scaling, (**A**) CU = 5 (very quiet), (**B**) CU = 15 (quiet), (**C**) CU = 25 (medium loud), (**D**) CU = 35 (loud), CU = 45 (very loud). As the loudness category increased, the measurement data increasingly fell within the 5th to 95th percentile of the control group. The low-performer data (VSB 20) was predominantly between the 25th and 75th percentile of all patient data.

The average sound pressure levels of the measurement data attributed to the loudness category ‘5 = very quiet’ (Fig. 3A) tended to be higher than the 95th percentile of the control group, with the exception of the frequency range ‘1 kHz’. The mean sound pressure levels attributed to the loudness category ‘15 = quiet’ (Fig. 3B) were predominantly between the mean value and the 95th percentile of the control group. The mean levels attributed to the loudness categories ‘25 = medium loud’, ‘35 = loud’ and ‘45 = very loud’ (Fig. 3C, D, E) were predominantly between the mean value and the 5th percentile of the control group. Particularly in the loudness category ‘45 = very loud’, the spread of the data was reduced compared to the aforementioned categories.

### PROM (SSQ and IOI-HA)

**Fig. 4:**
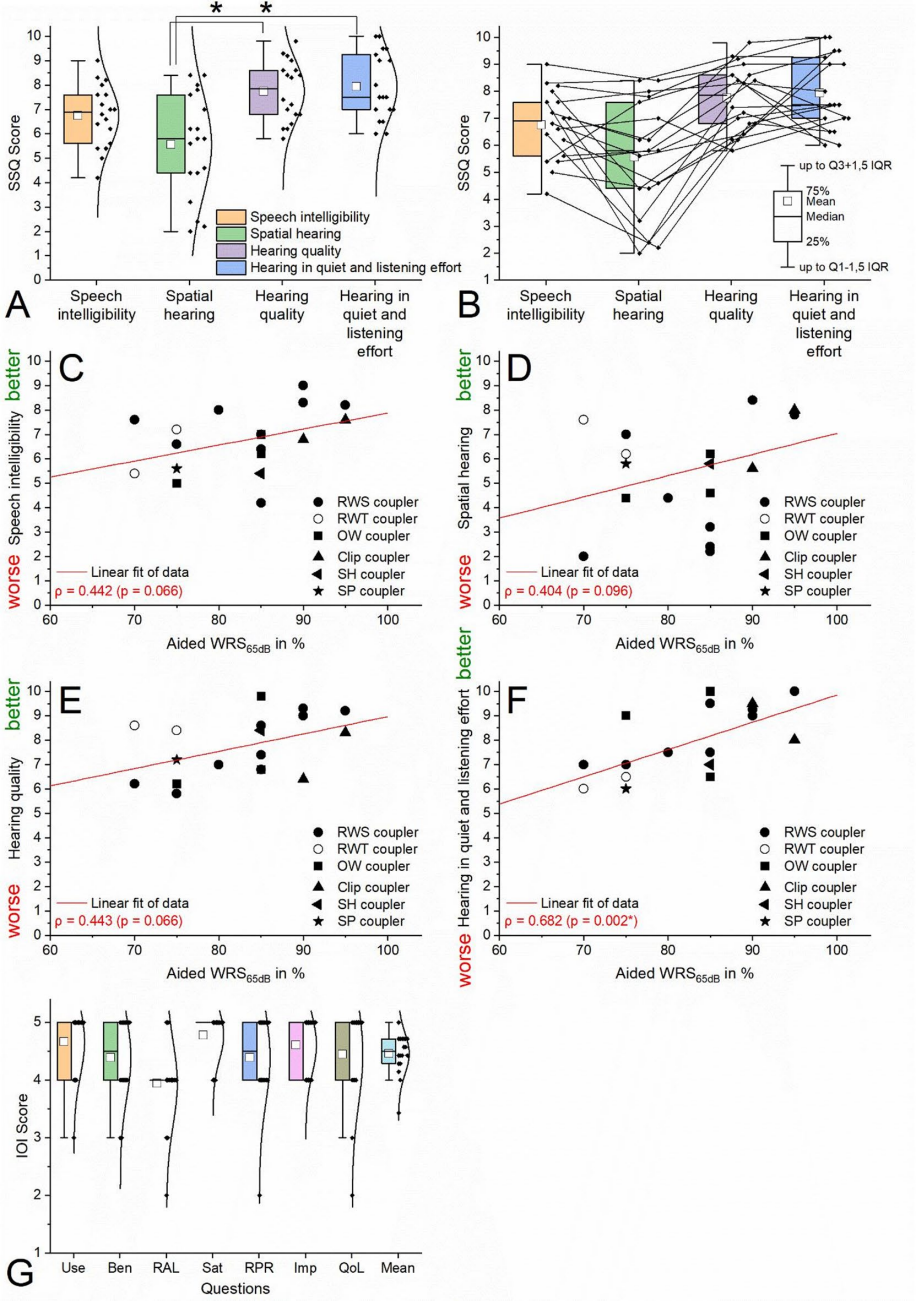
Results of PROM measurements. (**A**): SSQ, significantly better mean results for ‘hearing quality’ and ‘hearing in quiet and listening effort’ compared to ‘spatial hearing’ (*p* < 0.05), results of ‘speech intelligibility’ in between the highest and lowest results. (**B**): individual case analysis of the SSQ, tendentially, subjects showed comparable performances from subscale to subscale, especially in the extreme ranges. (**C** to **F**): Correlations between the subscales of the SSQ and postoperative speech understanding. Significant correlation between ‘hearing in quiet and listening effort’ and aided WRS_65dB_. (**G**): IOI-HA: excellent results in all questions as well as in the mean. Daily use (Use), Benefit (Ben), residual activity limitations (RAL), satisfaction (Sat), residual participation restrictions (RPR), impact on others (Imp), quality of life (QoL) * significant results, *p* < 0.05.

The SSQ measurements (Fig. 4A) showed acceptable results in all subscales. The lowest average results were found in the ‘spatial hearing’ subscale (5.6 ± 2.1 (SD), minimum: 2.0, maximum: 8.4). ‘Hearing in quiet and listening effort’ (7.9 ± 1.4 (SD), minimum: 6.0, maximum: 10.0) as well as ‘hearing quality’ (7.7 ± 1.2 (SD), minimum: 5.8, maximum: 9.8) showed the highest average results with a statistically significant difference from the subscale mentioned before (Welch-ANOVA: *p* < 0.05, Games-Howell post-hoc test: both *p* < 0.01, ω²=0.248, large effect). Average ‘speech intelligibility’ subscale (6.8 ± 1.3 (SD), minimum: 4.2, maximum: 9.0) was in between the highest and lowest subscales. Individual analysis of the SSQ (Fig. 4B) for all patients showed that the extreme ranges (high or low scores outside of the 25th to 75th percentile) tended to show continuity across all subscales. Correlations between the subscales of the SSQ and postoperative speech understanding (WRS_65dB_) were calculated and illustrated (Fig. 4C to F) using fitting functions as shown in Fig. 2. A strong and significant correlation with postoperative speech intelligibility was observed for the subscale ‘Hearing in quiet and listening effort’ (Fig. 4F, ρ = 0.682, *p* = 0.002). All other subscales showed moderate correlations but narrowly missed statistical significance.

The IOI-HA (Fig. 4C) showed excellent results across all questions and on average (4.5 ± 0.4 (SD), minimum: 3.4, maximum: 5.0). The lowest average score (3.9 ± 0.7 (SD), minimum: 2, maximum: 5) was for question 3: ‘Think again about the situation where you most wanted to hear better. When you use your present hearing aid(s), how much do you still have in that situation’. The highest average score (4.8 ± 0.4 (SD), minimum: 4, maximum: 5) was for question 4: ‘Considering everything, do you think your present hearing aid(s) is worth the trouble’.

## Discussion

A mean WRS_65dB_ of 81.5 ± 9.0% (SD) was achieved in the analyzed patient group. The results are within the range of previous studies in which an average WRS_65dB_ of 65 to 90% was reported^2,4,6,7,14–16^. The range for the WRS_65dB_ determined in this study was 60 to 95%. The study aimed to identify predictors for the audiological outcome. Understanding significant predictors supports optimized treatment planning and patient counselling. In addition, it facilitates the interpretation of postoperative outcomes and ultimately contributes to improving the overall quality of care.

### Value of cochlear capacity

Cochlear reserve is a key determinant of postoperative speech intelligibility following vibroplasty^2,6^. At this point, it is necessary to consider which measure represents cochlear capacity best when evaluating the likelihood of treatment success. The most commonly used metric in the literature is the averaged BC threshold across 0.5, 1, 2, and 4 kHz (PTA4). In recent years, it has become increasingly evident that the manufacturer-defined BC_PTA4_ indication limits are overestimated. Based on technical performance characteristics, including maximum power output (MPO) and a defined dynamic range (30–35 dB), Rahne and Plontke proposed a more restrictive recommendation for the indication range^1^. For the VSB, an averaged BC_PTA4_ threshold of 47 dB or 52 dB appears to represent a more realistic upper limit.

In the present study, all patients had BC_PTA4_ thresholds within this restricted indication range, suggesting an adequate cochlear reserve across the cohort. Nonetheless, a considerable variability in postoperative speech intelligibility was observed, with WRS_65dB_ values ranging from 60% to 95% (Δ = 35%). The averaged BC_PTA4_ threshold displayed a strong and statistically significant correlation with the postoperative WRS_65dB_ (see Fig. 2B). This finding contrasts with the results of Müller et al^6^., who reported no such correlation in a cohort of 23 patients—all of whom exhibited a preoperative WRS_max_ of ≥ 95%. This selection criterion may have limited the variability needed to detect such an association.

An alternative parameter for characterizing cochlear reserve is the preoperative WRS_max_, which has proven to be an excellent predictor of postoperative outcome in both hearing aid and cochlear implant users^17,18^. In our study, WRS_max_ ranged from 60% to 100% and correlated significantly with WRS_65dB_ (see Fig. 2H). This finding further supports its predictive value in vibroplasty.

### Value of coupling efficiency

One key factor contributing to the discrepancy between WRS_max_ and WRS_65dB_ is the coupling efficiency (CE). Postoperatively, this can be quantified by the difference between BC and Vibrogram thresholds. Reference values for Vibrogram measurements are based on temporal bone studies. These studies used coupling of the FMT to the long process of the incus and showed an expected variability of ± 10 dB. However, precise calibration data for other coupling methods remain unavailable. Under optimal conditions—i.e., ideal coupling to the long incus process—a difference of 0 dB across all frequencies would be expected, indicating perfect mechanical energy transfer. Positive values indicate an unfavorable coupling.

In our study, the average CE (PTA4) was 12.1 ± 9.6 dB (SD). In 16 patients (80%), CE values were below 20 dB, which has been identified as a critical threshold for favorable outcomes^6^. Patients included in our study displayed WRS_max_ values ranging from 60% to 100%, with a mean WRS_max_ of 88.0 ± 12.3% (SD). Importantly, even among patients with CE values exceeding 15 dB (*n* = 3), satisfactory speech intelligibility between 75% and 90% was achieved. An inverse relationship between CE and WRS_65dB _has also been demonstrated in a multicentric study by Müller et al^6^..

In our cohort, various coupling modalities were analyzed. It is well documented from both clinical and experimental studies that coupling quality differs significantly between coupling techniques and couplers^2,7^. In particular, RW coupling is known to result in poorer coupling efficiency compared to ossicular chain coupling. This effect is particularly evident in the low-frequency range and remains a surgical challenge. Even with the use of modern spherical soft couplers, this issue has only been partially addressed. One critical factor affecting coupling quality—and thereby hearing outcomes—is the preload applied to the RW membrane^19–22^. This parameter currently cannot be objectively assessed intraoperatively and therefore relies on the surgeon’s experience and subjective evaluation.

Consequently, intraoperative assessment of coupling quality with real-time electrophysiological measures is highly promising. This includes the recording of evoked acoustic potentials, particularly in the context of RW coupling^8,23^. Such techniques may reduce coupling-related variability and improve the consistency of audiological outcomes over time.

### Value of threshold-related parameters

Another important outcome parameter for assessing the effectiveness of vibroplasty is the EG, which quantifies the extent to which cochlear hearing loss can be compensated by the implant^24^. Historically, functional gain was commonly used as a fitting metric. However, this parameter is not suitable for evaluating BC hearing loss compensation in mixed or conductive hearing loss. In these cases, air–bone gap closure contributes to the improvement and thus confounds interpretation^24^. In contrast, the EG—derived from free-field thresholds—is a clinically established tool for audiometry-based implant fitting.

In our cohort, the EG demonstrated only a moderate but non-significant correlation with WRS_65dB_ (see Fig. 2E). At this point, limitations in the measurement of free-field thresholds should be acknowledged. In addition to intrinsic microphone noise, compression and feedback suppression algorithms may affect threshold accuracy. Individual patient tolerance can also play a role. Consistent with previous studies, a frequency-dependent pattern of the EG was observed. EG values were poorer (higher) in the low-frequency range and better (lower) in the mid- and high-frequency ranges^2,7,15^.

It is also well established that the EG is influenced by the type of vibroplasty. RW coupling is associated with poorer EG values, particularly at low frequencies, compared to ossicular coupling^2,7,15^. These differences partly reflect the quality of mechanical coupling but also underscore the reduced low-frequency transmission efficiency of the VSB. This technical limitation should be carefully considered when evaluating candidacy for implantation, particularly in patients with low-frequency hearing impairment^2^.

### Value of categorical loudness scaling

The application of loudness scaling to characterize suprathreshold auditory dynamics has proven effective in both CHAs and implantable hearing aid (Cochlear Implant (CI)) fitting. However, to date, no data have been published on its application in the fitting of AMEIs. In our study, loudness scaling revealed that for the loudness category “quiet” (15 CU), 70–75% of data points within the 1–2 kHz range fell within the reference range. For the “medium” category (25 CU), 90–95% of values at 1 and 2 kHz were within reference range. As expected, a complete compensation of loudness was not achieved at frequencies ≥ 4 kHz, likely due to limited patient tolerance for high-frequency amplification. At 0.5 kHz, the fitting algorithm often reduces gain by approximately 10 dB to prevent upward masking of speech-relevant acoustic cues. This effect is reflected in the “quiet” category at this frequency.

In our cohort, only one patient (ID 20) was identified as a low performer, with a WRS_65dB_ below 70% (WRS_65dB_ of ID 20 = 60%). Consequently, stratified evaluation of loudness scaling by performance group (high performance ≥ 70%, low performance < 70%) was not feasible. Notably, this patient’s loudness scaling data already fell within the reference range for the “quiet” category.

In all cases, the initial fitting of the VSB was based on Vibrogram thresholds. Using these data, the default setting was based on DSL [i/o]. Subsequent fine-tuning considered the aided WRS_65dB_, threshold data and the subjective perception. Loudness scaling was recorded exclusively within the framework of this study and did not guide the clinical fitting process.

As the literature lacks data on loudness-based fitting of AMEIs, future studies could aim to approximate the loudness scaling of normal-hearing listeners during the fitting process at multiple time points. Changes in other outcome parameters (PROMs and audiological measures) could then be analyzed in relation to these loudness scaling data to evaluate the impact of loudness-based fitting on outcomes.

### Value of patient-reported outcome measures

In addition to the audiological assessment of treatment success, subjective evaluation using standardized and validated PROMs should always be included^25^. For treatment with AMEIs, instruments originally developed for hearing aid users are still applied. Using the IOI-HA^26^, 89% (benefit (normative mean (NM) = 3.8^26^), residual activity limitations (NM = 3.5^26^), quality of life (NM = 3.8^26^)), 94.5% (daily use (NM = 4.2^26^), residual participation restriction (NM = 3.7^26^)) and 100% (satisfaction (NM = 4.0^26^), impact on others (NM = 3.8^26^)) of our patients included in the study achieved results exceeding the normative values reported for conventional hearing aid fittings^26^. Only one patient (ID 13) scored on average below the normative mean range (3.8)^26^, despite achieving aided speech intelligibility (WRS_65dB_) scores of 85% respectively. The mean score across all subscores in our data was 4.5. When compared with two studies in the literature including 19^27^ and 23^14^ patients, mean scores were 4.1^27^ and 4.5^14^. Reported subscores ranged from 3.8 (item: benefit^27)^ to 5.0 (item: daily use^27)^ with most of the items being on average above 4.0^14,27^.

Regarding the Speech, Spatial and Qualities of Hearing Scale (SSQ), mean normative values for normal-hearing younger adults (items: speech (8.7), spatial (8.8) and quality (9.9)) and older adults (items: speech (7.2), spatial (7.4) and quality (8.4))^28^ have been reported. For hearing impaired individuals without hearing rehabilitation^29^, mean values are lower (items: speech (5.5), spatial (5.2), qualities (6.8)). Our mean results (speech (6.8), spatial (5.6), quality (7.7)) fell between those of normal-hearing^28^ and unaided hearing impaired^29^ groups. In comparison, two further studies including 10^30^ and 22^31^ patients reported the following subscores: speech (5.8^31^and 9.5^30^), spatial (5.0^31^ and 7.5^30^), quality (5.2^31^ and 8.0^3^).

In our data, aided WRS_65dB_ showed moderate correlations with the subdomains ‘speech intelligibility’, ‘spatial hearing’, and ‘hearing quality’. A strong and statistically significant correlation was found for ‘hearing in quiet and listening effort’ (see Fig. 4). Although the moderate correlations narrowly missed statistical significance, the results suggest meaningful relationships. In future studies, PROMs may therefore be considered as valuable predictors within the framework of comprehensive outcome analyses.

### Limitations

Other important factors influencing the coupling efficiency have been identified in experimental studies. However, parameters such as coupling position, coupling stability, and alignment cannot be assessed in a standardized manner in clinical settings. There were therefore not available as potential predictors in the present analysis. Additionally, Vibrogram thresholds may be influenced by several variables, including the condition of the middle ear, the external auditory canal, tympanic membrane reconstruction, and annular ligament preload. These factors can consequently affect the evaluation of CE.

Individual patient history was also not systematically considered. One potentially relevant factor is the duration of inadequately compensated hearing loss. Patients with mixed or conductive hearing loss frequently undergo multiple ear surgeries and experience recurrent otorrhea, during which hearing aids cannot be worn. Evidence from cochlear implant research^32^ suggests that this parameter may significantly contribute to the variability of auditory outcomes. Another relevant limitation is the small sample size. The population eligible for and receiving active middle ear implants (AMEI) remains limited. As a result, single-center studies invariably yield small cohorts. A comprehensive multicenter evaluation would therefore be preferable. Overall, the data exhibited substantial variability, which affected the statistical analysis. This issue is consistent with findings reported in other studies on AMEI outcomes.

## Conclusion

This study provided a comprehensive analysis of audiological and patient-reported outcomes following VSB implantation as first part of a two-part publication series. Beyond conventional threshold-based metrics, we included suprathreshold loudness scaling and validated PROMs to obtain a multidimensional understanding of treatment outcomes.

The average aided WRS_65dB_ was 81.5%, with considerable interindividual variability (60–95%), despite all patients meeting stringent cochlear reserve criteria. Strong correlations were observed between WRS_65dB_ and both BC_PTA4_ and unaided WRS_max_, underscoring the continued relevance of these parameters for outcome prediction. PROMs (SSQ12 and IOI-HA) supported the audiometric results and provided additional insight into subjective benefit, with moderate and strong correlation found between WRS_65dB_ and the SSQ subscales.

Overall, the results confirm that a combination of audiometric, suprathreshold, and subjective measures is essential for a holistic assessment of AMEI outcomes. In the second part of this two-part publication series, the data presented in this manuscript will serve as the basis for the AI-supported development of a predictive model for postoperative speech intelligibility.

## Materials and methods

The single-centerstudy was conducted between 1 st March 2023 and 28th February 2024 in a tertiary referral hospital (Department of Otorhinolaryngology, Head and Neck Surgery at the University Hospital Carl Gustav Carus at the Technische Universität Dresden) in accordance with the Declaration of Helsinki and according to the rules of good clinical practice, following the review and approval of the local ethics committee at the Technische Universität Dresden (BO-EK-51012023).

### Inclusion criteria

During a single appointment at the study site, adult VSB users with acquired hearing loss who were unilaterally implanted at the study site were examined. In all patients, the BC and air conduction (AC) threshold (measured across the frequencies 0.5, 1, 2, 4 kHz and calculated as pure-tone average (PTA4)) on the contralateral side (non-implanted side) was a maximum of 30 dB, so that this side did not need fitting with a hearing system. All included patients were implanted according to the international consensus guidelines^24^.

When the VSB was implanted, the floating mass transducer (FMT) was attached depending on the individual pathology. If the ossicular chain (OC) was intact and mobile, the FMT was coupled to the incus body (SP) or long process of the incus (LP) using the SP or LP coupler. The selection between SP or LP coupler was based on the surgeon’s preference, as there are no recommendations for the best possible incus coupling. In the case of an OC defect, present stapes superstructure and mobile footplate, the FMT was coupled to the stapes head with a clip coupler (SClip) or SH coupler. If the chain was fixed or the stapes superstructure was absent, the FMT was coupled to the round window (RW) membrane (RW softcoupler (RWS) or RW titanium coupler (RWT)). All couplers mentioned above are medical devices manufactured by MED-EL.

Demographic details on the patients included, information on the implantation period, coupling site, applied coupler and etiology of hearing loss are provided in Table 2.

### Exclusion criteria

Underage patients (age < 18 years) and/or patients who were unable to give informed consent and/or who were unable to follow the study instructions and/or who suffered from neurological/psychological disorders were excluded from participation in the study.

### Timing of data collection

The measurements of the patients were conducted once during the study period. All patients had received their initial fitting at least 3 months prior to the study measurements.

### Initial and follow-up fitting

Initial fitting was done 6 weeks after implantation using the DSL[i/o] fitting formula based on the Vibrogram thresholds. The subsequent fitting was performed 4 weeks later, based on patient report and the audiometric results.

### Measurements

Please refer to Supp. 1, Table A1 to Table A5 for the preprocessed raw data.

### Audiological measurements

The audiometric measurements were conducted in a soundproof room (DIN EN ISO 8253) by means of a clinical audiometer (AT900 or AT1000, Auritec GmbH, Hamburg, Germany).

### Threshold-based measurements

At the implanted side, the BC hearing threshold (frequencies between 0.125 kHz and 6 kHz) was measured using pure tone audiometry by means of BC headphones (KLH 96, CB-Elmec, Germany) and the FF threshold was measured using narrowband noise in the free-field (narrowband noise in FF, center frequencies between 0.25 and 8 kHz). To obtain the FF threshold, the contralateral ear was plugged with a conventional earplug (SNR = 27 dB) and covered with earmuffs (Peltor Optime III H540A, SNR = 35 dB, 3 M, Minnesota, USA). The most recent air conduction hearing threshold (AC, frequencies between 0.125 and 8 kHz) prior to implantation was obtained from the patient data record stored in the clinic’s information system of the study site.

The Vibrogram was measured by presenting the stimulus directly to the FMT. The thresholds were determined using the Hughson-Westlake method and the results were reported on a decibel scale. This measurement method references the excitation voltage of the FMT (coupling to the long process of the incus using an LP coupler) at the hearing threshold for a person with normal hearing. For the FMT, the basic data refer to the coupling at the long process of the incus.

The following outcome parameters were calculated from the threshold-based measurements described above:

For BC, AC, FF thresholds and VIB, the PTA was calculated across the frequencies 0.5, 1, 2, and 4 kHz (PTA4) and 1, 2, and 4 kHz (PTA3), respectively.

The effective gain (EG, PTA4 or PTA3) was calculated as the difference between the FF threshold and the postoperative BC.

The coupling efficiency (CE, PTA4 or PTA3) was calculated as the difference between the Vibrogram threshold and the postoperative BC.

The dynamic range (DR, PTA4 or PTA3) was calculated as the difference between the aided FF threshold and the maximum power output of the VSB (75dB (0.5 kHz), 83 dB(1.0 kHz), 90dB (2 kHz), 80dB (4 kHz)^1^.

### Speech intelligibility testing

Speech intelligibility with activated implant was determined using the Freiburg monosyllabic word test in quiet at 65 dB SPL in free-field (aided WRS_65dB_), with the contralateral ear plugged and covered as described above. Speech was presented towards the front of the patient’s head (0°). One patient was a low performer with speech intelligibility of only 60% (VSB 20). His individual data are highlighted separately in all graphs.

The most recent preoperative maximum unaided speech intelligibility (WRS_max_) determined using the Freiburg monosyllabic word test in quiet (sound presentation by means of headphones, masking of the contralateral ear by means of speech masking noise with variable level) was obtained from the patient data record stored in the clinic’s information system of the study site. The (unaided) WRS_max_ is – besides of BC_PTA4_ threshold - the relevant parameter for the cochlear reserve and defines the hearing restoration level that should be obtained also in aided condition and with optimal fitting of the device^6^.

### Categorical loudness scaling

The loudness function as a measure of the relationship between sound level and subjective hearing perception for the implanted ear was determined using the categorical loudness scaling according to ISO 16832:2006 (sound pressure level (SPL): 15 to 80 dB, frequency range: 0.5 to 6 kHz, loudness rating: 5 (‘very quiet’), 15 (‘quiet’), 25 (‘medium’), 35 (‘loud’), 45 (‘very loud’)). The contralateral ear was plugged and covered as described above. The reference values (mean, 5th and 95th percentile) of 22 normal subjects (age: 16–42 years, median: 25 years) were taken from ISO 16832:2006.

### Patient reported outcome measures (PROMs)

The International Outcome Inventory for Hearing Aids (IOI-HA)^26^ validated in German and the Speech, Spatial and Qualities of Hearing scale (SSQ 12)^33^ were used to determine the subjective benefit of VSB implantation. The surveys were conducted in context with the aforementioned audiometric testing.

### Calculations and statistical analyses of audiological measurements and PROM

All calculations and statistical analyses were performed by means of Microsoft Excel (Microsoft Corporation, Redmond, U.S.), Origin (OriginLab, Northampton, U.S.) and SPSS (IBM, Armonk, U.S.). All statistical tests were performed at the significance level of α = 0.05. When the text refers to a trend or tendency, the results are not significant (*p* ≥ 0.05).

The majority of the data **(**Figs. 1, 3 and 4) are displayed by means of boxplots with the following tendency and dispersion measures: mean, median, 25% (Q1) to 75% (Q3) percentile, lower whisker (Q1 −1.5x interquartile range (IQR)), upper whisker (Q3 + 1.5x IQR).

All post-hoc analyses of effect sizes for significant differences between data were performed using Hedge’s g*, which was estimated from Hedge’s g in analogy to Eq. 1. The effect sizes were interpreted according to the rules of Cohen (|g*|= 0.2, small effect; |g*| = 0.5, medium effect; |g*|=0.8, large effect).

$$\:\text{g}\text{*}\:\approx\:\:\text{g}\:\times\:(1-\frac{3}{4\left({\text{n}}_{1}+\:{\text{n}}_{2}\right)-9}$$

with.

g∗ Hedge’s g*.

g Hedge’s g.

n1 and n2 group sizes (in case of testing only one sample, n2 is excluded from the equation).

Equation 1 Formula for estimating Hedge’s g*.

### Audiological measurements and health-related quality of life (HRQOL) (IOI-HA, SSQ)

For each threshold-based parameter (BC, FF, VIB, EG, CE, DR) (Fig. 1), the means of the individual frequencies were analyzed for significant differences using univariate ANOVA. Levene’s test was used to test the homogeneity of variance of the data, which could be confirmed. Group combinations were compared post hoc using Tukey’s test for significant differences in individual means. The effect size was calculated using the omega square (ω²) and interpreted according to Cohen: 0.01 ≤ |ω²|< 0.06, low effect; 0.06 ≤ |ω²| < 0.14, moderate effect; |ω²| ≥ 0.14, large effect^34^. The means of PTA4 and PTA3 were analyzed for significant differences by means of paired t-test, effect sizes were calculated by means of Hedge’s g* according Eq. 1. The data were analyzed for mild (Q1 − 1.5 x IQR, Q3 + 1.5 x IQR) and strong (Q1 − 3.0 x IQR, Q3 + 3.0 x IQR) outliers.

Monotonic correlations between aided WRS_65dB_ and the above mentioned threshold-based parameters as well as patients’ age and unaided WRS_max_ were calculated using Spearman’s correlation coefficient (ρ, monotonic correlation) (Supp. 2, Table A6). The results for PTA4 (showing a better absolute value of ρ than PTA3 for all threshold-based parameters), patients’ age and unaided WRS_max_ were plotted (Fig. 2). In addition, linear fit functions were calculated (Fig. 2 and Supp. 2, Table A7).The correlation coefficients are interpreted according to Cohen: low/weak correlation |ρ or R | = 0.10, medium/moderate correlation |ρ or R| = 0.30, high/strong correlation |ρ or R| = 0.50^35^. An aided WRS_65dB_ of less than 70% was defined as the boundary between high performers (WRS ≥ 70%, HP) and low performers (WRS < 70%, LP). This threshold was derived from multicenter clinical data, that reported an average aided WRS_65dB_ of 72% with the current (third) generation of couplers^16^. Our data show one low performer (ID 20). A residual analysis was used to identify outliers.

The results of the categorical loudness scaling (Fig. 3) were plotted descriptively in box plots against the above-mentioned reference (ISO 16832:2006). The low performer (VSB 20) was labeled separately.

Mean differences between subscales of the SSQ (Fig. 4A) were tested for significance using a univariate analysis of variance (ANOVA). Levene’s test was used to test the homogeneity of variance of the data in the subscales based on their means. Welch ANOVA was used for analysis due to lack of homogeneity of variance. Group combinations were compared post hoc using the Games-Howell test for significant differences in individual means. The effect size was calculated using the omega square (ω²) and interpreted according to Cohen (see above). In addition, a graphical individual case analysis (Fig. 4B) was performed to track the continuity of the individual score across all subscales. Monotonic correlations between aided WRS_65dB_ and the above-mentioned subscales were calculated using Spearman’s correlation coefficient (ρ, monotonic correlation). In addition, linear fit functions were calculated (Fig. 4C to F).

The evaluation of the results of the IOI (Fig. 4C) was only descriptive (boxplots, means, standard deviations).

### **Use of** translation aids

During manuscript preparation, DeepL and GPT-4o were utilized to translate the manuscript into English.

## Supplementary Information

Below is the link to the electronic supplementary material.

Supplementary Material 1

Supplementary Material 2

## Data Availability

The processed raw data of all patients/subjects are attached in the appendix.

## Abbreviations

AC: air conduction
AMEI: active middle ear implant
BC: bone conduction
CE: coupling efficiency
CHA: conventional hearing aid
CHL: conductive hearing loss
CI: Cochlear Implant
COMcC: chronic otitis media with cholesteatoma
COMsC: chronic otitis media without cholesteatoma
CU: loudness category
DSL [i/o]: desired sensation level input/output
DR: dynamic range
EG: effective gain
FF: free-field
FMT: floating mass transducer
g*: Hedge’s g* (effect size)
HRQOL: health-related quality of life
IOI-HA: International Outcome Inventory for Hearing Aids
IQR: interquartile range
LP: long process of the incus
MAE: mean absolute error
MHL: mixed hearing loss
MPO: maximum power output
NM: normative mean
OC: ossicular chain
ω²: effect size of ANOVA
OWC/OW coupler: oval window coupler
PROM: patient-reported outcome measures
PTA: pure tone average
ρ: Spearman’s correlation coefficient
RW: round window
RWSC / RWS coupler: round window soft coupler
RWTC / RWT coupler: round window titanium coupler
SClip / SClip coupler: stapes Clip coupler
SD: standard deviation
SH: stapes head
SSH / SH coupler: stapes SH coupler
SP: short process of the incus
SPC / SP coupler: short process coupler
SPL: sound pressure level
SSQ: Speech, Spatial and Qualities of Hearing scale
VIB: Vibrogram
VSB: Vibrant Soundbridge
WRS: word recognition score
WRS_65dB_: aided speech intelligibility at 65 dB SPL
WRS_max_: maximum unaided speech intelligibility
