# Supplementary material for: Comprehensive analysis of predictors and outcomes following Vibrant Soundbridge implantation – part 1 of a prospective study
Source: Sci Rep. 2025 Oct 10;15:35403. doi: 10.1038/s41598-025-20966-y (PMC12514048; doi:10.1038/s41598-025-20966-y)
Supplement: Supplementary file 1 — Supplementary Material 1 [file 41598_2025_20966_MOESM1_ESM.pdf]

Supplemental File 1 – Tables

ID	Age	Bone conduction (BC) in dB HL						Vibrogram (Vib) in dB						Free Field (FF) threshold in dB HL						WRS 65dB aided in %	WRS max unaided in %
		0,5 kHz	1,0 kHz	2,0 kHz	4,0 kHz	PTA4	PTA3	0,5 kHz	1,0 kHz	2,0 kHz	4,0 kHz	PTA4	PTA3	0,5 kHz	1,0 kHz	2,0 kHz	4,0 kHz	PTA4	PTA3		
1	78	15	50	65	70	50,0	61,7	65	55	55	70	61,3	60,0	35	40	40	60	43,8	46,7	70	70
2	58	20	30	30	30	27,5	30,0	55	45	30	35	41,3	36,7	45	40	35	30	37,5	35,0	70	60
3	30	15	25	20	25	21,3	23,3	45	30	15	20	27,5	21,7	30	30	20	30	27,5	26,7	90	100
4	36	10	20	20	25	18,8	21,7	70	70	35	45	55,0	50,0	25	25	40	35	31,3	33,3	85	70
5	67	25	45	45	35	37,5	41,7	25	40	35	55	38,8	43,3	30	35	25	35	31,3	31,7	75	80
6	57	30	35	35	30	32,5	33,3	45	45	30	25	36,3	33,3	30	35	35	30	32,5	33,3	80	90
7	35	20	25	35	55	33,8	38,3	40	20	20	45	31,3	28,3	30	30	35	55	37,5	40,0	75	80
8	41	20	35	45	30	32,5	36,7	40	30	25	25	30,0	26,7	30	25	25	30	27,5	26,7	95	100
9	33	15	20	25	20	20,0	21,7	45	30	25	20	30,0	25,0	40	35	50	40	41,3	41,7	95	100
10	61	5	15	35	40	23,8	30,0	45	40	25	40	37,5	35,0	30	25	20	35	27,5	26,7	90	85
11	32	25	30	25	15	23,8	23,3	40	30	25	25	30,0	26,7	35	35	25	40	33,8	33,3	85	100
12	27	0	5	25	45	18,8	25,0	45	30	35	60	42,5	41,7	15	15	35	65	32,5	38,3	90	90
13	30	45	45	50	30	42,5	41,7	65	65	45	40	53,8	50,0	55	50	50	30	46,3	43,3	85	100
14	46	20	20	25	40	26,3	28,3	30	25	25	65	36,3	38,3	40	25	30	50	36,3	35,0	85	95
15	69	5	25	40	30	25,0	31,7	55	45	45	55	50,0	48,3	35	30	35	50	37,5	38,3	80	95
16	54	10	15	30	30	21,3	25,0	40	30	30	30	32,5	30,0	35	30	30	30	31,3	30,0	75	100
17	37	5	20	40	65	32,5	41,7	35	35	40	70	45,0	48,3	35	35	50	50	42,5	45,0	85	90
18	28	5	5	5	0	3,8	3,3	35	15	10	5	16,3	10,0	40	35	55	50	45,0	46,7	85	100
19	53	25	35	35	50	36,3	40,0	55	60	60	70	61,3	63,3	30	30	35	50	36,3	38,3	75	75
20	68	25	35	30	40	32,5	35,0	55	45	40	50	47,5	45,0	35	35	35	45	37,5	38,3	60	90

Tab A1 Age and Hearing results I (raw data): bone conduction, vibrogram, free field threshold, preoperative unaided maximum speech intelligibility (WRSmax) measured with headphones, postoperative aided speech intelligibility at 65dB (WRS65) measured in free field conditions.

ID	Coupling Efficiency (Vib - BC) in dB						Effective Gain (FF - BC) in dB						VSB output in dB HL				Dynamic Range (VSB output - FF) in dB					
	0,5 kHz	1,0 kHz	2,0 kHz	4,0 kHz	PTA4	PTA3	0,5 kHz	1,0 kHz	2,0 kHz	4,0 kHz	PTA4	PTA3	0,5 kHz	1,0 kHz	2,0 kHz	4,0 kHz	0,5 kHz	1,0 kHz	2,0 kHz	4,0 kHz	PTA4	PTA3
1	50	5	-10	0	**11,3**	**-1,7**	20	-10	-25	-10	**-6,3**	**-15,0**	75	83	90	80	40	43	50	40	**43,3**	**44,3**
2	35	15	0	5	**13,8**	**6,7**	25	10	5	0	**10,0**	**5,0**	75	83	90	80	30	43	55	45	**43,3**	**47,7**
3	30	5	-5	-5	**6,3**	**-1,7**	15	5	0	5	**6,3**	**3,3**	75	83	90	80	45	53	70	60	**57,0**	**61,0**
4	60	50	15	20	**36,3**	**28,3**	15	5	20	10	**12,5**	**11,7**	75	83	90	80	50	58	50	40	**49,5**	**49,3**
5	0	-5	-10	20	**1,3**	**1,7**	5	-10	-20	0	**-6,3**	**-10,0**	75	83	90	80	45	48	65	55	**53,3**	**56,0**
6	15	10	-5	-5	**3,8**	**0,0**	0	0	0	0	**0,0**	**0,0**	75	83	90	80	45	48	55	45	**48,3**	**49,3**
7	20	-5	-15	-10	**-2,5**	**-10,0**	10	5	0	0	**3,8**	**1,7**	75	83	90	80	45	53	55	45	**49,5**	**51,0**
8	20	-5	-20	-5	**-2,5**	**-10,0**	10	-10	-20	0	**-5,0**	**-10,0**	75	83	90	80	45	58	65	55	**55,8**	**59,3**
9	30	10	0	0	**10,0**	**3,3**	25	15	25	20	**21,3**	**20,0**	75	83	90	80	35	48	40	30	**38,3**	**39,3**
10	40	25	-10	0	**13,8**	**5,0**	25	10	-15	-5	**3,8**	**-3,3**	75	83	90	80	45	58	70	60	**58,3**	**62,7**
11	15	0	0	10	**6,3**	**3,3**	10	5	0	25	**10,0**	**10,0**	75	83	90	80	40	48	65	55	**52,0**	**56,0**
12	45	25	10	15	**23,8**	**16,7**	15	10	10	20	**13,8**	**13,3**	75	83	90	80	60	68	55	45	**57,0**	**56,0**
13	20	20	-5	10	**11,3**	**8,3**	10	5	0	0	**3,8**	**1,7**	75	83	90	80	20	33	40	30	**30,8**	**34,3**
14	10	5	0	25	**10,0**	**10,0**	20	5	5	10	**10,0**	**6,7**	75	83	90	80	35	58	60	50	**50,8**	**56,0**
15	50	20	5	25	**25,0**	**16,7**	30	5	-5	20	**12,5**	**6,7**	75	83	90	80	40	53	55	45	**48,3**	**51,0**
16	25	15	0	0	**10,0**	**5,0**	25	15	0	0	**10,0**	**5,0**	75	83	90	80	40	53	60	50	**50,8**	**54,3**
17	30	15	0	5	**12,5**	**6,7**	30	15	10	-15	**10,0**	**3,3**	75	83	90	80	40	48	40	30	**39,5**	**39,3**
18	30	10	5	5	**12,5**	**6,7**	35	30	50	50	**41,3**	**43,3**	75	83	90	80	35	48	35	25	**35,8**	**36,0**
19	30	25	25	20	**25,0**	**23,3**	5	-5	0	0	**0,0**	**-1,7**	75	83	90	80	45	53	55	45	**49,5**	**51,0**
20	30	10	10	10	**15,0**	**10,0**	10	0	5	5	**5,0**	**3,3**	75	83	90	80	40	48	55	45	**47,0**	**49,3**

Tab A2 Hearing results II (raw data): coupling efficiency, effective gain, VSB maximum output hearing level (Rahne & Plontke 2022), dynamic range (Rahne et al. 2016).

ID	Loudness Scaling																								
	ISO 5					ISO 15					ISO 25					ISO 35					ISO 45				
	0,5 khz	1 kHz	2 kHz	4 kHz	6 kHz	0,5 khz	1 kHz	2 kHz	4 kHz	6 kHz	0,5 khz	1 kHz	2 kHz	4 kHz	6 kHz	0,5 khz	1 kHz	2 kHz	4 kHz	6 kHz	0,5 khz	1 kHz	2 kHz	4 kHz	6 kHz
**1**	42	30		61	51	67	65		73	64	86	90		85	76	94	99		95	88	99	100		105	98
**2**	60	37	41	43	61	77	59	64	66	85	92	76	83	84	103	100	88	93	93	109	105	96	98	98	109
**3**	41	31	27	22	30	59	54	54	38	47	76	74	76	55	61	86	86	88	73	71	96	95	96	91	79
**4**	40	37	34	33	30	55	49	46	45	43	69	62	60	59	55	82	76	76	74	67	94	92	93	91	78
**5**	35	34	40	37	35	58	53	57	49	47	77	70	72	62	59	88	83	85	80	70	96	94	95	99	81
**6**	29	32	37	49	44	59	59	67	75	63	82	81	90	93	80	93	92	99	99	89	98	97	99	100	97
**7**	25	21	27	57	56	48	39	45	63	65	69	57	63	69	75	83	74	78	79	87	94	92	93	88	99
**8**													-												
**9**	37	19	30	20	33	53	38	48	40	50	69	57	65	58	66	82	74	80	77	80	94	92	94	94	94
**10**	39	32	43	47	62	68	60	62	66	78	89	82	78	81	92	97	93	88	91	100	99	97	96	97	106
**11**	37	34	33	37	28	55	55	58	62	56	71	73	78	82	78	84	86	89	93	90	94	95	97	98	97
**12**													-												
**13**	45	41	33	35	34	56	46	39	47	47	68	55	47	60	59	81	69	64	76	70	94	85	84	92	81
**14**	34	28	31	37	43	55	52	55	46	51	73	71	75	59	64	86	85	88	83	87	95	95	96	110	115
**15**													-												
**16**	27	22	32	23	36	50	47	54	47	55	70	68	74	68	72	84	83	86	82	85	94	94	95	94	95
**17**	45	37	46			65	62	71			81	82	89			90	91	97			95	96	98		
**18**	51	29	52	40	44	64	47	65	57	57	77	63	76	72	71	87	78	87	84	83	97	92	97	95	94
**19**	36	27	42	55	54	58	41	65	72	71	77	56	83	87	86	88	76	92	95	95	96	96	97	100	101
**20**	26	21	31	45	35	48	49	51	57	50	68	72	70	69	63	82	87	83	82	10	94	95	94	93	75

Tab A3 Hearing results III (raw data): loudness scaling.

ID	SSQ 17																	Mean Speech Intelligibility	Mean Spatial Hearing	Mean Hearing Quality	Mean Additional Questions
	1_1	1_2	1_3	1_4	1_5	2_1	2_2	2_3	2_4	2_5	3_1	3_2	3_3	3_4	3_5	4_1	4_2				
1	5	6	4	7	5	8	8	9	5	8	9	9	7	9	9	9	3	5,4	7,6	8,6	6
2	6	8	6	10	8	2	2	2	2	2	7	6	8	8	2	9	5	7,6	2	6,2	7
3	8	8	8,5	9	8	8,5	8	9	7,5	9	9	9	8,5	10	10	9,5	9	8,3	8,4	9,3	9,25
4	6	8	7	7	4	2	3	3	3	1	5	7	8	8	9	8	7	6,4	2,4	7,4	7,5
5	6	5	10	10	5	6	7	10	4	4	8	9	10	7	8	8	5	7,2	6,2	8,4	6,5
6	11	8	8	8	5	11	4	5	0	2	6	8	7	7	7	8	7	8	4,4	7	7,5
7	7	5	8	3	2	5	6	4	3	4	5	7	3	8	8	10	8	5	4,4	6,2	9
8	9	8	9	9	6	7	7	6	11	8	10	9	9	9	9	10	10	8,2	7,8	9,2	10
9	7	7	7	8	9	9	9	8	7	7	6,5	8	9	9	9	8	8	7,6	8	8,3	8
10	8	7	6	9	4	7	7	5	5	4	8	8	8	8	0	10	9	6,8	5,6	6,4	9,5
11	9	7	7	8	4	3	3	4	4	2	9	8	8	9	9	10	9	7	3,2	8,6	9,5
12	9	9	8	10	9	9	10	9	7	7	9	9	9	9	9	10	8	9	8,4	9	9
13	4	7	8	2	0	1	1	1	4	4	3	5	9	9	8	7	8	4,2	2,2	6,8	7,5
14	5	8	9	9	4	5	5	7	5	9	9	10	10	10	10	10	10	7	6,2	9,8	10
15	-																				
16	7	8	5	6	7	7	7	7	7	7	6	7	6	4,5	5,5	7	7	6,6	7	5,8	7
17	2	4	8	5	8	7	7	6	4	5	9	8	7	9	9	11	3	5,4	5,8	8,4	7
18	7	7	7	5	5	5	5	3	5	5	7	7	7	6	7	8	5	6,2	4,6	6,8	6,5
19	7	4	5	6	6	5	7	7	5	5	6	6	8	8	8	7	5	5,6	5,8	7,2	6
20	-																				

Tab A4 SSQ 17 (german version): raw data and section means.

ID	IOI							
	1	2	3	4	5	6	7	Mean
1	5	4	3	5	4	4	5	4,3
2	4	4	4	4	5	5	5	4,4
3	5	5	4	5	4	5	5	4,7
4	5	4	4	5	5	5	5	4,7
5	5	5	5	5	5	5	5	5,0
6	5	4	4	4	5	5	4	4,4
7	5	5	5	5	4	4	5	4,7
8	5	5	4	5	5	5	2	4,4
9	5	5	4	5	5	5	4	4,7
10	3	4	4	5	4	5	4	4,1
11	5	5	4	5	4	5	4	4,6
12	5	4	4	5	5	5	5	4,7
13	4	3	2	4	4	4	3	3,4
14	4	5	4	5	5	5	5	4,7
15	-							
16	4	5	5	5	4	4	5	4,6
17	5	4	4	5	4	4	5	4,4
18	5	3	3	5	5	4	5	4,3
19	5	5	4	4	2	4	4	4,0
20	-							

Tab A5 IOI-HA (german version): raw data and mean.
